# LONELINESS IN COMMUNITY-DWELLING US OLDER LATINX ADULTS

**DOI:** 10.1093/geroni/igac059.1695

**Published:** 2022-12-20

**Authors:** David Camacho, Kelly Pacheco, Todd Becker, Cary Reid, Elaine Wethington

**Affiliations:** University of Maryland, Baltimore, Norwalk, California, United States; New York University, New York City, New York, United States; University of Maryland, Baltimore, Baltimore, Maryland, United States; Weill Cornell Medicine, New York City, New York, United States; Cornell, Ithaca, New York, United States

## Abstract

Loneliness is a critical public health problem yet little is known about older Latinx’s experiences with loneliness. We conducted narrative interviews (Cultural Formulation Interview), with 17 Spanish-speaking Latinx (60 years+) living with loneliness (based on 3-item Loneliness scale) in New York or Los Angeles. Interviewers were bilingual (English/Spanish) gerontological social workers with training in qualitative methods, culturally-sensitive clinical research and elicitation of “sensitive data”. Our thematic analysis was informed by Biopsychosocial and Minority Stress Models, and our gerontological clinical experience with Latinx groups. Most participants immigrated from Mexico, Dominican Republic, Ecuador or Uruguay and lived in the US for more than 20 years. Interviews ranged from 60 to 120 minutes. Most participants described comorbid depression or pain. A substantial majority (82%) had never discussed feelings of loneliness with family or friends. No participant reported seeking professional help to address loneliness. Other identified themes demonstrated how cultural and contextual factors shape loneliness: 1) Descriptions and interpretations rooted in familism and views of aging (“it seems I no longer matter to my children”); 2) perceived causes e.g. discrimination, family conflict, bereavements, immigrant stress, violence; 3) religious and cognitive coping to mitigate loneliness (“I pray that God gives me strength, “I befriend loneliness”); and 4) psychosocial functioning impact ( “there is no reason to live”). Our findings support development of culturally congruent loneliness prevention and treatment practices for older Latinx adults. Future quantitative research should explore the association of cultural and minority stress factors related to loneliness in diverse samples of older Latinx.

